# The Effect of Intrauterine Administration of Growth Hormone on IVF Success Rate in Recurrent Implantation Failure Women: A Randomized Clinical Trial

**DOI:** 10.5812/ijpr-153636

**Published:** 2024-12-16

**Authors:** Fatemeh Amirkhanloo, Mohammad Javanbakht, Sarah Lotfi, Ghazal Sahraiyan, Razieh Akbari, Elham Feizabad, Shima Rahimi, Mahbod Ebrahimi, Firouzeh Akbari Asbagh, Fateme Davari Tanha

**Affiliations:** 1 Department of Obstetrics and Gynecology, Yas Hospital Complex, Tehran University of Medical Sciences, Tehran, Iran; 2 Nephrology and Urology Research Center, Clinical Science Institute, Baqiyatallah University of Medical Sciences, Tehran, Iran; 3 Department of Infertility, Qom University of Medical Sciences, Qom, Iran; 4 Vali-E-Asr Reproductive Health Research Center, Family Health Research Institute, Tehran University of Medical Sciences, Tehran, Iran

**Keywords:** Growth Hormone, Intrauterine Administration, Endometrial Receptivity, RIF

## Abstract

**Background:**

The positive effects of growth hormone (GH) on the endometrium, including increased endometrial blood supply and enhanced expression of cytokines associated with endometrial receptivity, have been noted. However, data on the effect of GH on the endometrium remain limited.

**Objectives:**

This study aimed to investigate the effect of intrauterine administration of GH on the IVF success rate in women with recurrent implantation failure (RIF).

**Methods:**

This randomized double-blind clinical trial was conducted on 60 infertile women under 40 years old with a Body Mass Index (BMI) below 30 kg/m², all diagnosed with RIF—defined as at least three failed pregnancies after transferring a minimum of four good-quality embryos due to unknown causes. Women with uterine malformations, Asherman syndrome, cavity-distorting lesions, severe endometriosis, or other underlying diseases were excluded. After six days of estrogen therapy, transvaginal ultrasound (TVS) was performed to measure and compare the thickness and quality of the endometrium. Participants were divided into two groups. In the intervention group, 10 units of GH were administered using an IUI catheter positioned one centimeter above the cervical os. Study outcomes included changes in endometrial thickness (ET) and quality, as well as pregnancy rates. Primary endpoints were changes in ET and quality, while secondary endpoints were pregnancy rates. Adverse drug responses were also evaluated.

**Results:**

The mean age was 34.96 ± 4.04 years, and the mean BMI was 24.89 ± 2.91 kg/m², with no significant differences in baseline variables between the study groups. The average ET on the 8th day of the cycle was 5.38 ± 0.96 mm in the intervention group and 5.20 ± 0.80 mm in the control group, showing no significant difference (P = 0.467). The ET on the day of initiating progesterone was 7.60 ± 1.03 mm in the intervention group and 7.40 ± 0.60 mm in the control group, with no significant difference (P = 0.264). The odds ratio for achieving a high-quality endometrium was 2.37 (95% CI 0.80 - 6.98, P = 0.116) for the GH group compared to the non-GH group. The odds ratio for achieving a clinical pregnancy was 3.06 (95% CI 0.54 - 17.37, P = 0.205) for the GH group compared to the non-GH group. Two cases of cervicitis were reported in the GH group.

**Conclusions:**

Intrauterine administration of GH appears to enhance endometrial receptivity in women with RIF.

## 1. Background

Implantation, a complex process, involves the attachment and penetration of the embryo into the deep layers of the uterine wall. The success rate of this mechanism depends on numerous factors (1, 2). During the implantation stage, the dividing fertilized ovum attaches to the uterine wall. It is possible that the concentration of substances in the follicular fluid correlates with the degree of ovum maturation, the fertilization success rate, and the extent of embryo implantation into the uterus (3). 

Proper implantation plays a crucial role in the success of in vitro fertilization (IVF) cycles and signifies an effective connection between the embryo and the endometrium (2). In many cases, implantation does not occur despite the presence of a high-quality embryo (4). Hormones and other regulatory substances involved in the implantation process are either secreted locally within the ovary (such as steroid hormones and cytokines) or produced externally and subsequently enter the follicle (5). 

The concentration of substances in the follicular fluid may correlate with ovum maturation, fertilization success, and embryo implantation in the uterus. Among these substances, growth hormone (GH) is notable, as some studies have highlighted its role in implantation (5-7). Growth hormone is a non-glycosylated polypeptide chain secreted by pituitary gland cells in all vertebrates, exhibiting a wide range of biological activities (8). It has significant and diverse therapeutic applications in medicine, believed to stem from its beneficial effects (9). Beyond its effects on blood circulation and local production, GH influences the endometrium and plays a role in the function and maintenance of the corpus luteum (9). 

Previous studies have demonstrated that the administration of GH during ovarian stimulation enhances IVF success rates, particularly in women with poor ovarian response. Recently, a hypothesis has emerged regarding the effect of GH on the endometrium and its potential benefits for women experiencing recurrent implantation failure (RIF) due to a thin endometrium (4, 10). Recurrent implantation failure is defined as the absence of pregnancy after at least four good-quality embryo transfers across a minimum of three cycles in women under 40 years of age (10). While the positive effects of GH on oocyte quality have been reported in several studies (11), its influence on uterine receptivity and implantation remains unclear (12). 

## 2. Objectives

This study aimed to assess the effect of intrauterine administration of GH on the IVF success rate in women with RIF. 

## 3. Methods

This randomized double-blind clinical trial was conducted on 60 infertile women diagnosed with RIF who were referred to the IVF department at a university-based hospital affiliated with Tehran University of Medical Sciences, Tehran, Iran, from October 2023 to March 2024. 

The study received approval from the Review Board of Tehran University of Medical Sciences (IR.TUMS.MEDICINE.REC.1402.285). The trial protocol was registered with the Iranian Registry of Clinical Trials under registration number IRCT20091012002576N34. Standardized procedures were followed to obtain written informed consent from participants. 

Women under 40 years of age with a Body Mass Index (BMI) of less than 30 kg/m² and diagnosed with RIF—defined as at least three failed pregnancies after transferring a minimum of four good-quality embryos due to unknown causes—were included in the study. Exclusion criteria included women using corticosteroids or glucose control agents, those with uncontrolled hypothyroidism, liver disease, CNS tumors, uterine malformations, Asherman syndrome, cavity-distorting lesions, severe endometriosis, or severe male infertility, as well as those unwilling to participate. 

The random allocation rule method assigned eligible women into two equal groups. On the second day of the menstrual cycle, transvaginal ultrasound (TVS) (4.5 - 7 MHz vaginal probe, Sono Line G-40, Siemens, Germany) was performed for all participants to measure endometrial thickness (ET). If ET was < 3 mm, 6 mg estradiol (Abu Reihan Pharmaceutical Company, Iran) was prescribed daily for 6 days. 

The trial utilized a random allocation rule method, assigning patients to either group in a 1:1 ratio. The randomization was conducted by an independent statistician. The study employed a double-blinding methodology, the participants and the analyzer were blinded to the treatment assignments. 

On the eighth day of the cycle, a TVS was performed to reassess ET and quality. The maximum ET was measured from one endometrial–myometrium interface to the corresponding interface. An additional 2 mg was added to the estradiol dose (totaling 8 mg). In the intervention group, 10 units of GH were administered using an insulin syringe and an IUI catheter positioned 1 cm above the cervical os. 

On the 12th day of the cycle, all patients underwent TVS. If ET exceeded 7 mm, 400 mg of vaginal progesterone (Abu Reihan Pharmaceutical Company, Iran) was initiated every 12 hours. For cases where ET was less than 7 mm, the 8 mg estradiol regimen was continued until ET reached 7 mm or more. Five-day embryos (grade A or B) were transferred after 6 days of progesterone application using a COOK catheter under ultrasound guidance. If pregnancy occurred, 400 mg of vaginal progesterone was continued daily until eight weeks of pregnancy. 

Patient-related variables measured included age, BMI, number of previous pregnancies, history of underlying diseases and their treatments, and chronic drug use. The primary endpoint was the change in ET and quality, while the secondary endpoint was the pregnancy rate. Additional adverse drug responses were also evaluated. 

The sample size was estimated at 30 subjects per group based on data from a prior study (13), ensuring a significant difference at a 5% alpha level with 80% power, calculated using GPower 3.1 software. This resulted in a total sample size of 56 subjects (n = 56). 

All analyses were conducted using SPSS version 23 software, with a significance level set at less than 0.05. The Kolmogorov-Smirnov test was applied to evaluate the distribution of continuous variables. Descriptive statistics for continuous variables are presented as means and standard deviations (SD), while frequencies and percentages are reported for categorical variables. *t*-tests and Mann-Whitney U tests were used to compare continuous variables between groups, while Fisher's exact test or the chi-square test was applied for categorical variables. An intention-to-treat analysis was performed, including all participants initially assigned after randomization. 

## 4. Results

**Figure 1.**
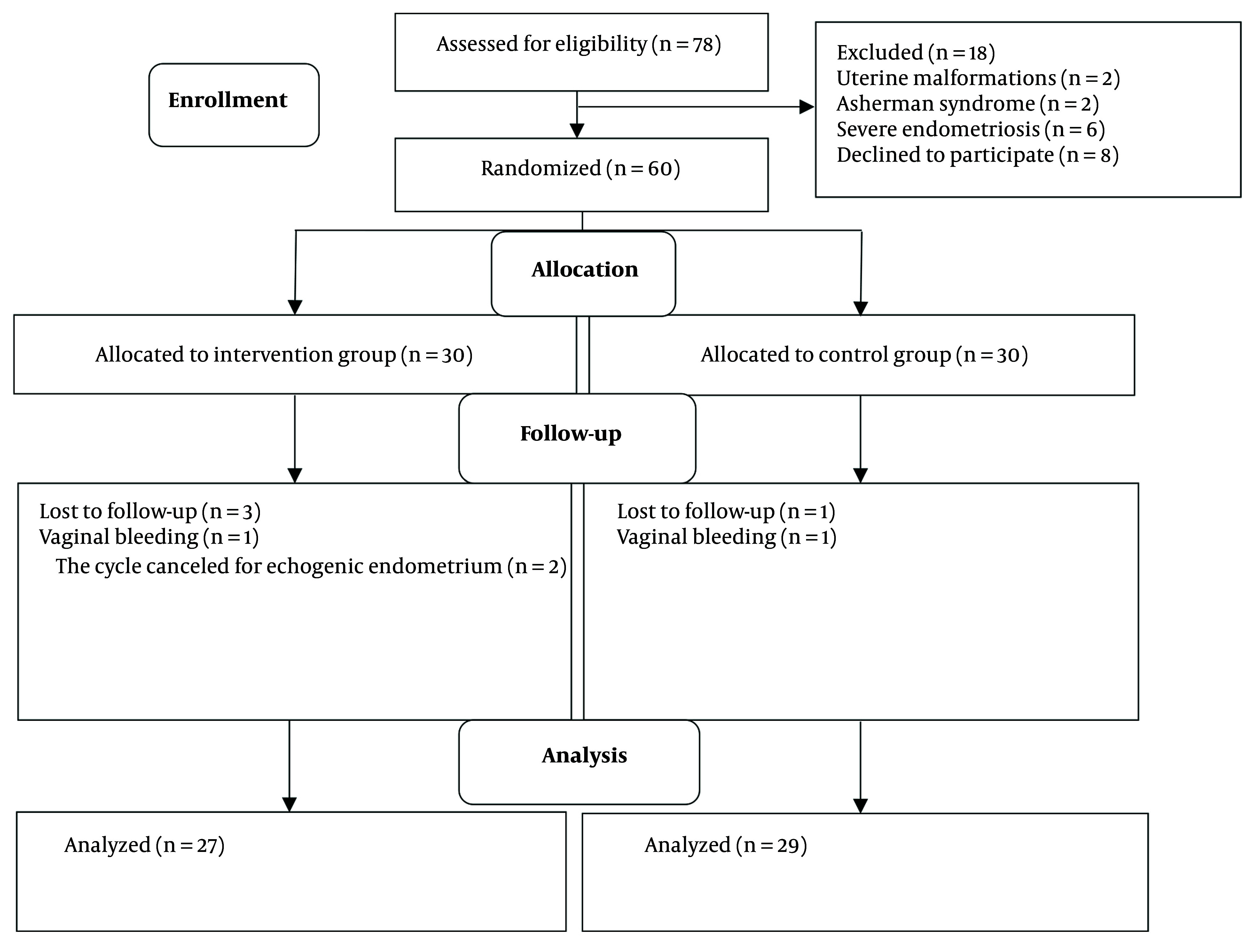
The flow diagram of the study

Out of the 60 patients examined, three women in the intervention group were lost to follow-up (one due to vaginal bleeding and two due to echogenic endometrium), and one woman in the control group was lost due to vaginal bleeding. Ultimately, 27 women in the intervention group and 29 in the control group completed the study (Figure 1). 

Demographics and baseline clinical parameters are summarized in Table 1. The mean age was 34.96 ± 4.04 years, and the mean BMI was 24.89 ± 2.91 kg/m², with no significant differences in baseline variables between the study groups. The average duration of infertility among participants was 6.55 ± 4.04 years, and 83.9% of patients had primary infertility. The most common cause of infertility was unexplained, accounting for 53.6%. 

**Table 1.** Comparison of General Information on Recurrent Implantation Failure Women Between the Two Groups ^a^

Variables	Intervention Group (n = 27)	Control Group (n = 29)	P-Value
**Age (y)**	34.19 ± 4.72	35.69 ± 3.19	0.173
**Body Mass Index (kg/m** ^**2**^ **)**	24.99 ± 3.13	24.81 ± 2.78	0.831
**Follicle stimulating hormone (mg/dL)**	6.67 ± 2.42	7.43 ± 1.78	0.218
**Anti-mullerian hormone (mg/dL)**	3.01 ± 2.53	4.15 ± 2.22	0.094
**History of diabetes**	1 (3.7)	2 (6.9)	0.596
**History of hypertension**	2 (7.4)	1 (3.4)	0.511
**History of hypothyroidism**	5 (18.5)	2 (6.9)	0.182
**History of surgery**	9 (31)	9 (33.3)	0.854
**Infertility duration (y)**	7.5 ± 4.9	5.7 ± 2.8	0.110
**Infertility type **			0.053
Primary	20 (74.1)	27 (93.1)	
Secondary	7 (25.9)	2 (6.9)	

^a^ Values are expressed as men ± SD or No. (%).

The average ET on the 8th day of the cycle was 5.38 ± 0.96 mm in the intervention group and 5.20 ± 0.80 mm in the control group, showing no significant differences (P = 0.467). Additionally, the ET on the day of starting progesterone was 7.60 ± 1.03 mm in the intervention group and 7.40 ± 0.60 mm in the control group, with no significant differences (P = 0.264). 

The quality of the endometrium on the day of starting progesterone was classified as type A in 15 cases (55.5%) in the intervention group and 10 cases (34.5%) in the control group, with no significant difference (P = 0.116). The odds ratio for having a high-quality endometrium was 2.37 (95% CI 0.80 - 6.98) for the GH group compared with the non-GH group. 

Additionally, the number of clinical pregnancies in the intervention group was 5 cases (18.5%), compared to 2 cases (6.9%) in the control group, with no significant difference (P = 0.205). The odds ratio for achieving a clinical pregnancy was 3.06 (95% CI 0.54 - 17.37) for the GH group compared with the non-GH group (Table 2). 

**Table 2.** Comparison of Study Outcomes on Recurrent Implantation Failure Women Between the Two Groups

Variables	Intervention Group (n = 27)	Control Group (n = 29)	P-Value
**ET at 8th day of cycle (mm)**	5.38 ± 0.96	5.20 ± 0.80	0.467
**ET at day of starting progesterone (mm)**	7.60 ± 1.03	7.40 ± 0.60	0.264
**Clinical pregnancy**	5 (18.5)	2 (6.9)	0.189
**Quality of A endometrium**	15 (55.5)	10 (34.5)	0.005

Abbreviations: ET, endometrial thickness.

### 4.1. Adverse Events

Standard surveillance procedures for adverse drug reactions were implemented in both groups to ensure consistent detection and reporting. In the GH group, two cases of cervicitis were reported, both of which were successfully managed, with no subsequent recurrence observed.

## 5. Discussion

Type A quality of the endometrium during embryo transfer was significantly higher in the intervention group (intrauterine administration), which aligns somewhat with the findings of previous studies involving the subcutaneous use of GH (13, 14). Various factors influence the success of IVF, with the quality of the endometrium being the most crucial for achieving proper implantation. Some studies have suggested that GHs can assist in this area, particularly in patients who have experienced multiple IVF failures (15, 16). 

The key factors in the implantation process are endometrial receptivity and embryo quality, and GHs have shown potential benefits in this context (13-16). Growth hormone has been shown to enhance endometrial blood supply and increase the expression of cytokines associated with endometrial receptivity. It has also been found to improve ET and increase the concentration of vascular endothelial growth factor (16, 17). 

Researchers have noted that GH combined with progesterone can significantly mitigate factors contributing to poor ovarian function in patients undergoing IVF. Studies have demonstrated that ET, embryo quality, the number of transferred embryos, implantation rates, and clinical pregnancy outcomes were significantly higher in the GH and progesterone group compared to the progesterone-only group (18). 

The authors suggested that incorporating human GH into an IVF cycle briefly improves live birth rates. It was also found that GH increases the likelihood of retrieving higher-quality ova, although it does not necessarily increase the chances of embryo transfer in these individuals. The findings of the present study regarding the improvement in uterine wall quality following intrauterine GH administration are consistent with the findings of these studies (18, 19). 

In the study by Hosseini Aghdam et al., conducted on infertile women with a history of frozen embryo transfer (FET) failure and refractory thin endometrium, participants received intrauterine administrations of GH. The results indicated that ET and implantation rates improved following GH administration, leading to higher pregnancy rates (18). Similarly, our findings revealed comparable ET and an increased implantation rate after intrauterine GH administration in women experiencing RIF. 

In the present study, the proportion of type A endometrium in the intrauterine GH administration group was higher, consistent with the findings of Jiang et al. They compared the effects of intrauterine administration of granulocyte-colony stimulating factor (GCSF) with sub-endometrial administration of GH in 66 infertile women with IVF failure and thin endometrium. The results demonstrated that GCSF enhanced the implantation rate, while GH improved endometrial quality; consequently, both increased pregnancy rates (19). 

Evidence suggests that GH administration in women undergoing IVF treatment increases the density of FSHR, BMPR1B, LHR, and granulosa GHR receptors compared to patients of similar age and ovarian reserve who did not receive GH. Additionally, GH enhances ET, the success of embryo implantation, and the density and activity of GHR. Concomitant GH treatment has been associated with a significant rise in pregnancy rates (20). 

Keane et al. demonstrated that clinical pregnancy and live birth rates were significantly higher in women who underwent IVF with GH supplementation. The patient’s age, the quality of the transferred embryo, and the dosage and timing of GH administration were identified as the only independent and significant predictors of clinical pregnancy. Growth hormone increased the likelihood of clinical pregnancy by 3.42 times and the chance of live birth by 6.16 times (21). 

This study assessed the effect of intrauterine GH administration in FET cycles for women with RIF and indicated a trend toward increased implantation rates. To the best of our knowledge, this is the first clinical trial evaluating the effect of intrauterine administration of 10 units of GH in RIF patients undergoing HRT-primed FET cycles, showing improved grade A endometrial quality and a trend toward higher clinical pregnancy rates. However, due to the small sample size, the clinical pregnancy rate did not reach statistical significance. 

Despite its strengths, this study has limitations, including a small sample size and a brief follow-up period. The selection of a specific group of infertile women (those with RIF) and the single-center nature of the study limit its generalizability. Further research is needed to determine the effective dose and optimal method of GH administration, as well as to identify which groups of infertile women would benefit most from this treatment. 

### 5.1. Conclusions

Intrauterine administration of GH appears to facilitate communication between the endometrium and the embryo by promoting the regeneration and repair of endometrial receptors essential for implantation in women with RIF. However, further research is needed to establish the optimal route and dosage of GH for this purpose.

## Data Availability

The dataset presented in the study is available on request from the corresponding author during submission or after its publication. The data are not publicly available due to internal policy.

## Clinical Trial Registration Code:

This study is registered with the Iranian Registry of Clinical Trials (IRCT) under the code IRCT20091012002576N34.
